# Reply to Trosch et al. Comment on “Dashtipour et al. Dysphagia and Muscle Weakness Secondary to Botulinum Toxin Type A Treatment of Cervical Dystonia: A Drug Class Analysis of Prescribing Information. *Toxins* 2024, *16*, 442”

**DOI:** 10.3390/toxins17040191

**Published:** 2025-04-11

**Authors:** Khashayar Dashtipour, Han S. Lee, Aaron Ellenbogen, Rashid Kazerooni, Todd M. Gross, David A. Hollander, Conor J. Gallagher

**Affiliations:** 1 Department of Neurology/Movement Disorders, Loma Linda University, Loma Linda, CA 92354, USA; 2 Department of Neurology, The Permanente Medical Group, Greater Southern Alameda Area—San Leandro/Fremont, San Leandro, CA 94577, USA; han.neurology@gmail.com; 3 Quest Research Institute, Farmington Hills, MI 48334, USA; aellenbogen@comcast.net; 4 Revance Therapeutics, Nashville, TN 37203, USA; rashidkazerooni@gmail.com (R.K.); tgross@revance.com (T.M.G.); david.a.hollander.md@gmail.com (D.A.H.); cgallagher@revance.com (C.J.G.)

We thank Trosch et al. [1] for their interest in our recent publication entitled, “Dysphagia and Muscle Weakness Secondary to Botulinum Toxin Type A Treatment of Cervical Dystonia: A Drug Class Analysis of Prescribing Information”. In response to their Comment [1], we address the authors’ concerns grouped across four main themes: 1. cross-trial comparisons; 2. quantifying nanograms of core neurotoxin content; 3. data on higher doses of daxibotulinumtoxinA; 4. guidance techniques across the different studies. We will address their concerns in sequence.

First, while we agree that head-to-head studies are preferred, comparisons across different studies are common and often necessary to help inform clinical care before direct head-to-head studies are conducted [2]. Our analysis was not based on a random selection of studies but rather relied upon studies described in the Prescribing Information (PI) for each of the U.S.-registered botulinum toxin type A products. In this case, the PIs served as the most consistent source of data due to the rigor of pivotal registration trials and the systematic collection of adverse events. In addition, the PIs are ultimately one of the primary resources upon which clinicians rely to inform treatment decisions.

Second, to clarify for the authors, the amount of core neurotoxin in nanograms per unit of biological activity of daxibotulinumtoxinA can be found in two publications [3,4]. Earlier studies have published ELISA-derived nanogram amounts of 150 kDa botulinum toxin type A in the products of various manufacturers [5,6,7]. Their findings have been broadly consistent, and any small differences do not fundamentally change our conclusions.

Third, while 250U was the maximum dose of daxibotulinumtoxinA administered in the double-blind ASPEN Phase 3 studies, doses of up to 300U were studied in ASPEN-OLS, the Phase 3 Open-Label Study. Safety and efficacy data from this study have been presented in poster form, and the manuscript is currently under peer review. The dysphagia and muscle weakness rates observed at the 300U dose were low and, therefore, are supportive of the findings of our analysis. These findings are further supported by the recent real-world daxibotulinumtoxinA early experience program, which includes doses above 300U and in which low rates of dysphagia and muscle weakness were observed [8].

Finally, the question is raised by the authors regarding guidance techniques in clinical trials, citing that guidance was not used in Poewe 1998, and the use of guidance when injecting the sternocleidomastoid can help reduce the risk of dysphagia. We note that the U.S. abobotulinumtoxinA Phase 2 study, which has been both published [9] as well as presented in the label (and, thus, reflected in our analysis), also allowed the use of EMG, and the adverse event rates for the approved 500U dose in that study [9] were consistent with those of other studies reflected in the PI and our analysis. Additionally, both the main Phase 3 and open-label extension Phase 3 trials for incobotulinumtoxinA allowed for the use of EMG guidance [10,11].

We reiterate our rationale for utilizing registration trials presented in PIs as the most consistent source of data due to the rigor of a Phase 3 trial as well as the systematic method of adverse event collection. In doing so, we stand behind our findings, including the association between key adverse event rates and core neurotoxin nanogram content that was revealed as a part of the analysis. The findings have not been reported previously and may offer fresh insights that help guide clinical decisions in the use of botulinum toxin type A in the treatment of cervical dystonia.
